# Molecular Characterization of Parvovirus Strain GX-Tu-PV-1, Isolated from a Guangxi Turkey

**DOI:** 10.1128/MRA.00152-19

**Published:** 2019-11-14

**Authors:** Yanfang Zhang, Zhixun Xie, Xianwen Deng, Zhiqin Xie, Liji Xie, Minxiu Zhang, Sisi Luo, Qing Fan, Jiaoling Huang, Tingting Zeng, Sheng Wang

**Affiliations:** a Guangxi Key Laboratory of Veterinary Biotechnology, Guangxi Veterinary Research Institute, Nanning, Guangxi, China; KU Leuven

## Abstract

The aim of the current study was to determine the genomic sequence of parvovirus strain GX-Tu-PV-1, which was isolated from a turkey in Guangxi Province, South China. The analysis showed that the genome sequence of GX-Tu-PV-1 was 81.3% to ∼99.3% similar to those of other turkey parvoviruses (TuPVs) and 79.8% to ∼92.1% related to chicken parvovirus (ChPV). This study will help in understanding the epidemiology and molecular characteristics of parvovirus in turkeys.

## ANNOUNCEMENT

Parvovirus (family *Parvoviridae*) spp. that infect vertebrate hosts make up the subfamily *Parvovirinae*, while those that infect arthropods make up the subfamily *Densovirinae* (1–4). According to the latest classification by the International Committee on Taxonomy of Viruses (https://talk.ictvonline.org/taxonomy/), the *Parvovirinae* subfamily is divided into eight genera (5–8) (Amdoparvovirus, Aveparvovirus, Bocaparvovirus, Copiparvovirus, Dependoparvovirus, Erythroparvovirus, Protoparvovirus, and Tetraparvovirus). Chicken parvovirus (ChPV) and turkey parvovirus (TuPV) are classified as members of the new genus Aveparvovirus (Galliform aveparvovirus 1).

In this study, viral DNA was extracted from tracheal and cloacal swabs from 12 turkeys using the EasyPure viral DNA/RNA kit (Transgen, Beijing, China). TuPV was identified by PCR, using primers designed to target the conserved region (nonstructural [NS] gene, 561 bp) first (1). The two positive samples were confirmed by partial sequencing of the nonstructural 1 (NS1) gene using primers NSF and NSR (Table 1). BLAST (https://blast.ncbi.nlm.nih.gov/Blast.cgi) analysis revealed 98.0% to ∼100% nucleotide (nt) sequence identity to the TuPV 1030 strain (GenBank accession number KM598418). Based on an alignment of the sequences of three TuPV isolates deposited in GenBank, the specific pairs of primers (Table 1) were then designed to amplify the genome of one positive TuPV sample named GX-Tu-PV-1. The gel-electrophoresed PCR products were purified by using the AxyPrep DNA gel extraction kit (Hangzhou, China), followed by cloning into the pMD-19T vector (TaKaRa, Japan). Sanger sequencing was performed on a DNA analyzer (Invitrogen, Guangzhou, China). The undetermined 5′ and 3′ terminal fragments were amplified using the 5′ rapid amplification of cDNA ends (RACE) system kit v.2 (TaKaRa) and reverse transcription-PCR with oligo(dT) primers, respectively. The amplified fragments were cloned into the pMD-19T vector (TaKaRa), and 4 and 8 clones of the 5′ and 3′ terminal regions, respectively, were sequenced. The sequences were obtained by assembling overlapping contigs, followed by editing using the EditSeq and MegAlign programs of DNAStar 7.0 green (DNAStar, Madison, WI).

**TABLE 1:** Primers used for PCR amplification of the turkey parvovirus genome

Primer^*a*^	Sequence (5′–3′)^*b*^	Nucleotide positions^*c*^
F1-1	CTGCTGAGCTGGTAAGATGG	395–414
R1-2	TCTTCCCGACTGACTAGATT	724–743
R1-3	CCCCCATGATACATTTTGCT	1751–1770
F2-1	TTCTAATAACGATATCACTCAAGTTTC	1841–1867
R1-4	AACCAGTATAGGTGGGTTCC	2192–2211
R1-1	TTTGCGCTTGCGGTGAAGTCTGGCTCG	2375–2401
F3-1	CAAGCCGCCATTGTGTTTGT	3575–3594
R2-2	GTATTGKGTYTGGTTTTCAG	3659–3678
R2-1	AAGTCWAKRTAATTCCATGG	3694–3713
R3-2	GTCCCTGTCAAGTCATTAGAG	3858–3878
R3-1	TTAATTGGTYYKCGGYRCSCG	5005–5025
NSF	TTCTAATAACGATATCACTCAAGTTTC	1841–1867
NSR	TTTGCGCTTGCGGTGAAGTCTGGCTCG	2375–2401

a Primers F1-1/R1-1, F1-1/R1-2, F1-1/R1-3, and F1-1/R1-4 were used to amplify the first fragment, F2-1/R1-1 and F2-1/R2-2 were used to amplify the second fragment, and F3-1/R3-1 and F3-1/R3-2 were used to amplify the third fragment of the turkey parvovirus (GX-Tu-PV-1) genome. Primers NSF to NSR were used to amplify the partial NS sequence.

b The sequences of the primers were designed according to the sequences of three other known ChPV/TuPV strains (GenBank accession numbers GU214704, GU214706, and NC_024454).

c The positions of primers located in the genome are shown according to the U.S. TuPV isolate (TuPV 1030).

**FIG 1:**
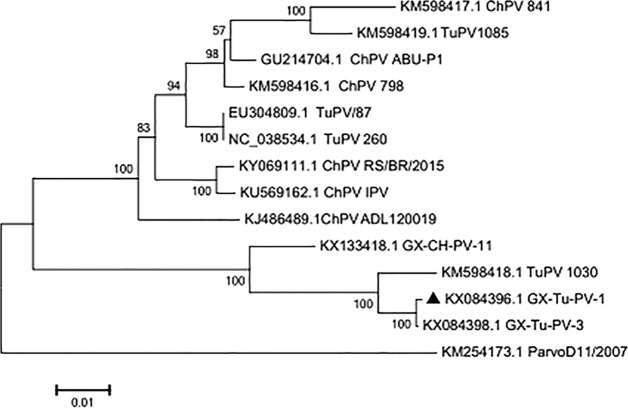
Neighbor-joining (NJ) tree of the genomic sequences of GX-Tu-PV-1 and 13 other ChPV/TuPV isolates. GenBank accession numbers follow the names of the ChPV/TuPV strains. The numbers near the branches indicate the confidence level calculated by bootstrapping (*n* = 1,000). The length of each pair of branches represents the distance between sequence pairs. The scale bar represents 0.01-nt substitutions per site. ▲, GX-Tu-PV-1.

The DNA sequence of the obtained isolate was 4,642 bp long, with an A+T content of 57.1% and G+C content of 42.9%. The entire genome of GX-Tu-PV-1 consists of three open reading frames (ORFs). ORF1 and ORF2 encode a nonstructural (NS) protein, which is involved in viral replication, and the major capsid proteins (VP1 and VP2), respectively. ORF3 encodes a putative protein, NP1, of which the function is unclear (9–13). The genetic diversity of GX-Tu-PV-1 was explored using phylogenetic analyses using ClustalW (http://www.clustal.org/clustal2/) and MEGA 7.0 (14). It showed that the genome sequence of GX-Tu-PV-1 was 81.3% to ∼99.3% related to TuPVs (GenBank accession numbers NC_038534, EU304809, KM598418, KM598419, and KX084398) and 79.8% to ∼92.1% related to ChPV strains (GenBank accession numbers KM598417, GU214704, KM598416, KY069111, KU569162, KJ486489, KX133418, and KM254173) (Fig. 1).

The sequence data of the GX-Tu-PV-1 strain will facilitate research on the epidemiology and evolutionary biology of parvoviruses in China.

### Data availability.

The genome sequence of GX-Tu-PV-1 was deposited in GenBank under the accession number KX084396.
